# Supplementary material for: Peripheral Blood Biomarkers Predictive of Efficacy Outcome and Immune-Related Adverse Events in Advanced Gastrointestinal Cancers Treated with Checkpoint Inhibitors
Source: Cancers (Basel). 2022 Jul 31;14(15):3736. doi: 10.3390/cancers14153736 (PMC9367581; doi:10.3390/cancers14153736)
Supplement: Supplementary file 1 [file cancers-14-03736-s001.zip › Table S1.pdf]

Table S1. Immune-related adverse events categorized by different treatment types

Immune-related adverse events (categories)	Total events (N=260)	Drug target(s)	P value (for total events)	CTCAE Grade 1 (N=170)	CTCAE Grade 2 (N=58)	CTCAE Grade 3-4 (N=32)	P value (for grade 3-4 events)
Skin	52	PD-1	**0.034**	18	5	2	0.411
		PD-L1		5	2	0	
		PD-1+CTLA-4		13	5	2	
Rheumatology	35	PD-1	0.544	8	5	6	0.356
		PD-L1		4	1	3	
		PD-1+CTLA-4		7	0	1	
Pulmonary	9	PD-1	0.050	2	2	0	NA
		PD-L1		2	1	0	
		PD-1+CTLA-4		1	1	0	
Gastrointestinal	21	PD-1	**<0.001<**	2	2	1	0.117
		PD-L1		3	2	0	
		PD-1+CTLA-4		6	3	2	
Endocrine	49	PD-1	**<0.001**	15	2	1	0.435
		PD-L1		4	0	0	
		PD-1+CTLA-4		20	6	1	
Hepatology	59	PD-1	0.650	25	7	4	0.050
		PD-L1		6	2	3	
		PD-1+CTLA-4		8	2	2	
Cardiology	6	PD-1	0.246	1	0	1	0.655
		PD-L1		1	0	0	
		PD-1+CTLA-4		1	1	1	
Nephrology	7	PD-1	0.499	1	1	1	0.655
		PD-L1		0	1	0	
		PD-1+CTLA-4		1	1	1	
Others	22	PD-1	**0.013**	8	2	0	NA
		PD-L1		0	1	0	
		PD-1+CTLA-4		8	3	0	

CTCAE, Common Terminology Criteria for Adverse Event; NA, not applicable. P values were indicated in bold when statistical results were significant.
