# Supplementary figures and images for: Peripheral Blood Biomarkers Predictive of Efficacy Outcome and Immune-Related Adverse Events in Advanced Gastrointestinal Cancers Treated with Checkpoint Inhibitors
Source: Cancers (Basel). 2022 Jul 31;14(15):3736. doi: 10.3390/cancers14153736 (PMC9367581; doi:10.3390/cancers14153736)

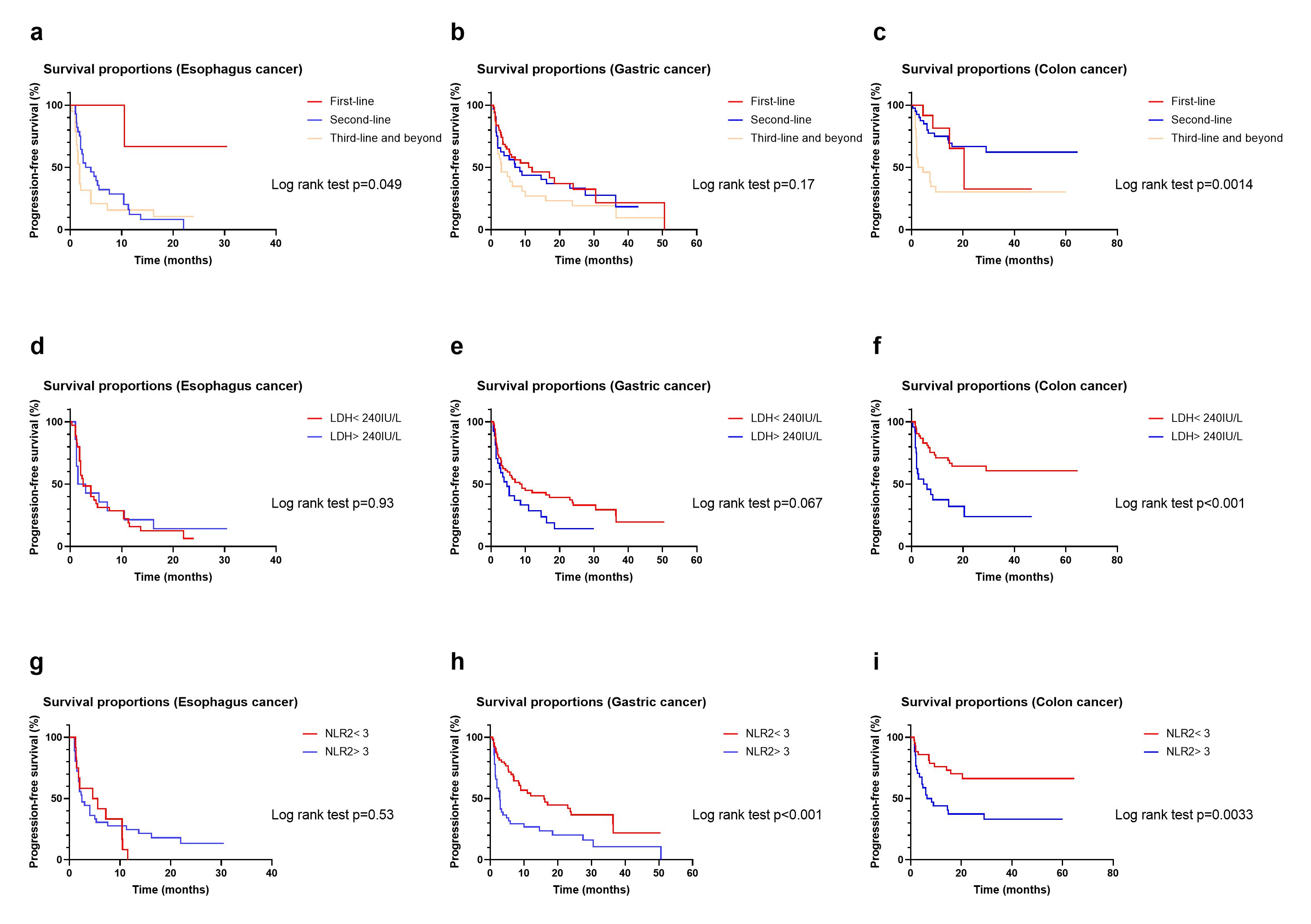

Supplement: Supplementary file 1 [file cancers-14-03736-s001.zip › Figure S1.jpg]

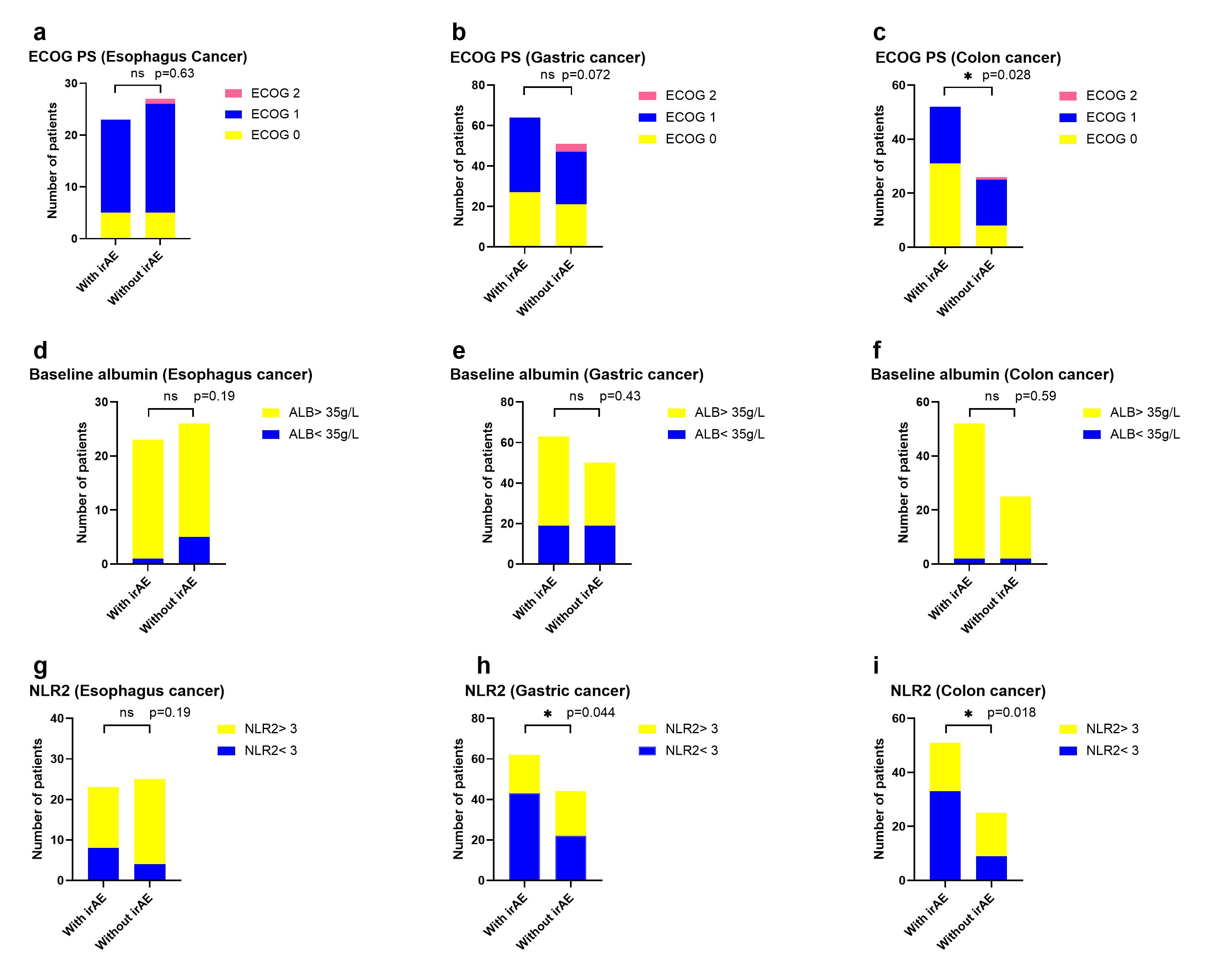

Supplement: Supplementary file 1 [file cancers-14-03736-s001.zip › Figure S2.jpg]

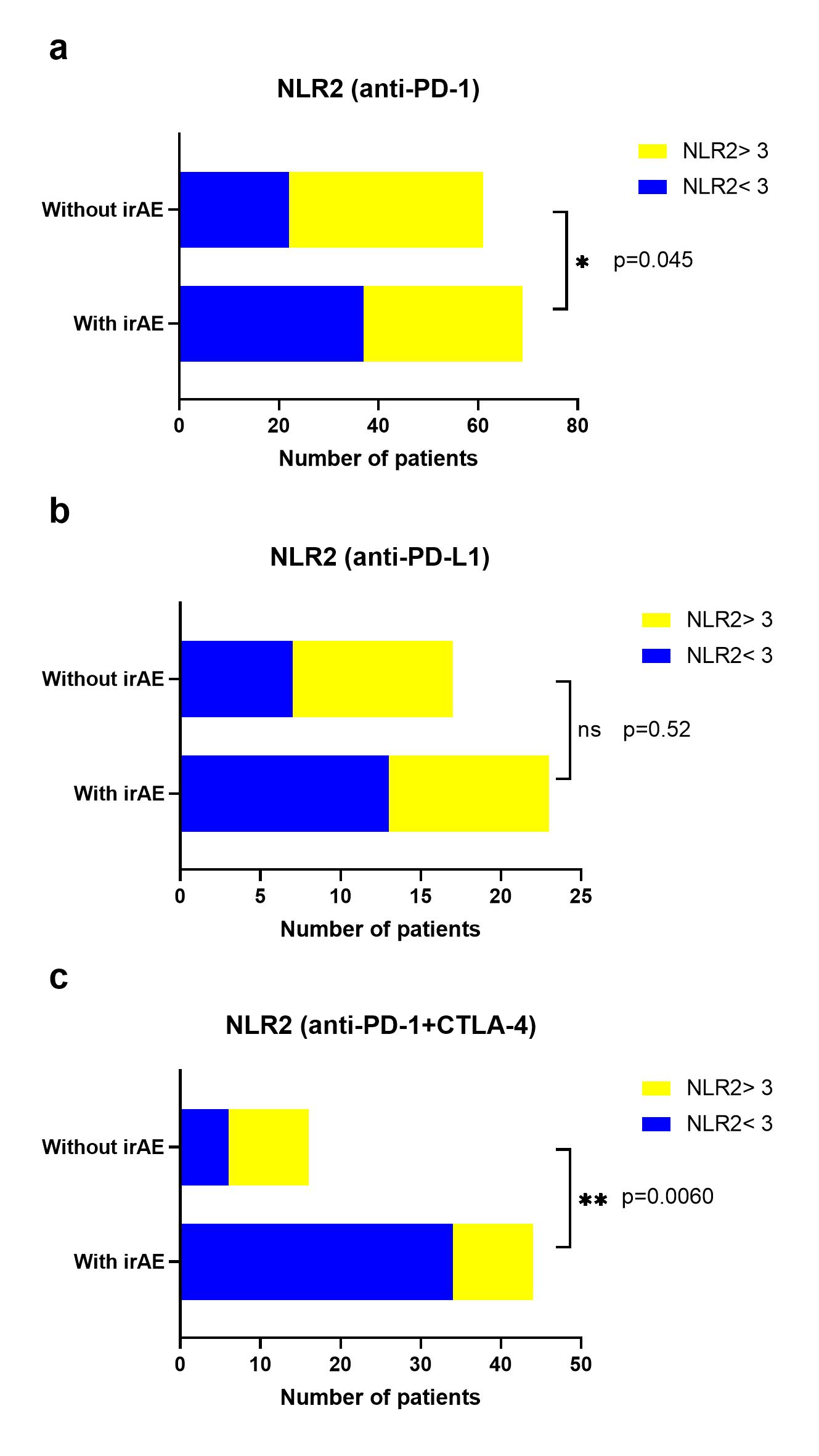

Supplement: Supplementary file 1 [file cancers-14-03736-s001.zip › Figure S3.jpg]

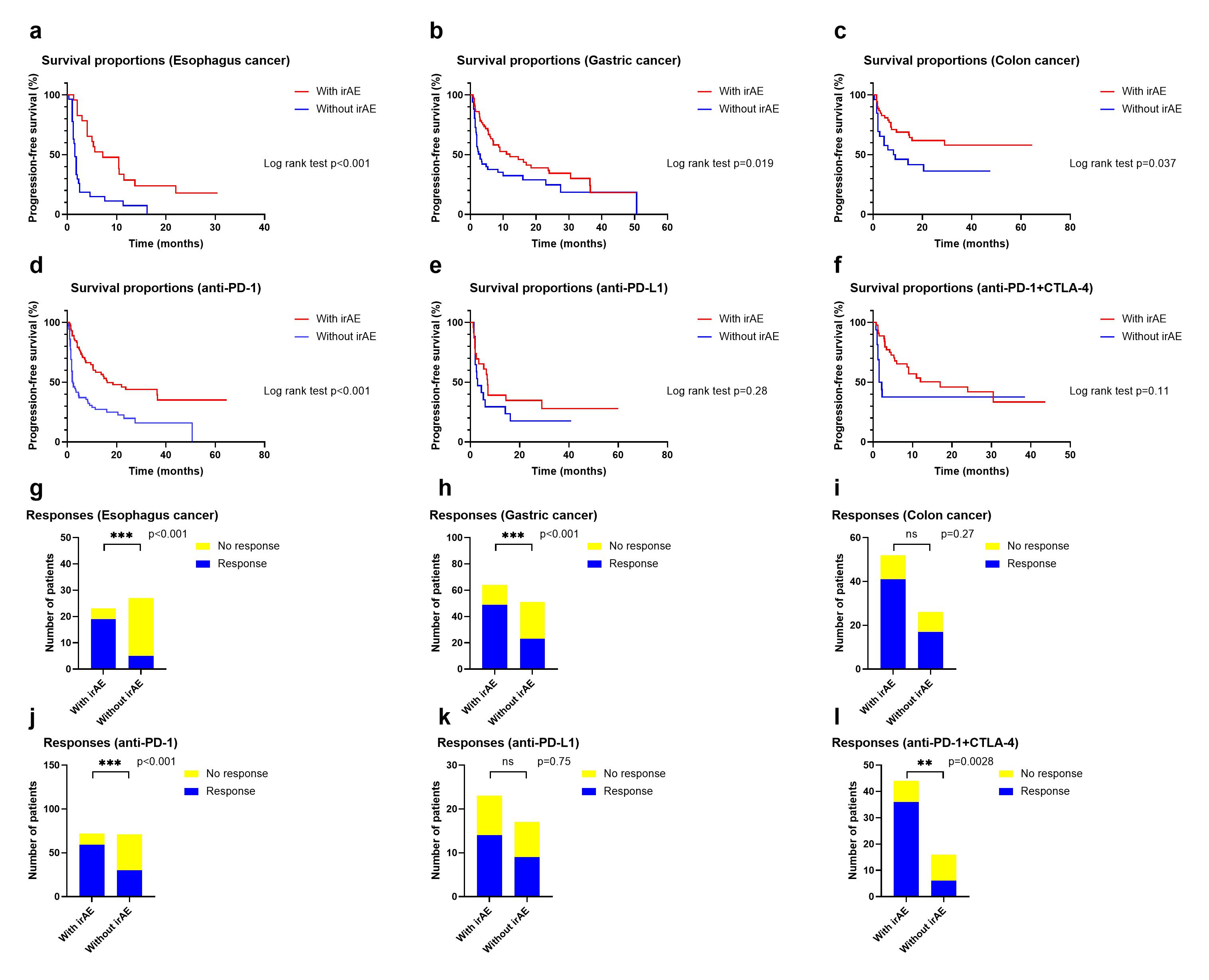

Supplement: Supplementary file 1 [file cancers-14-03736-s001.zip › Figure S4.jpg]

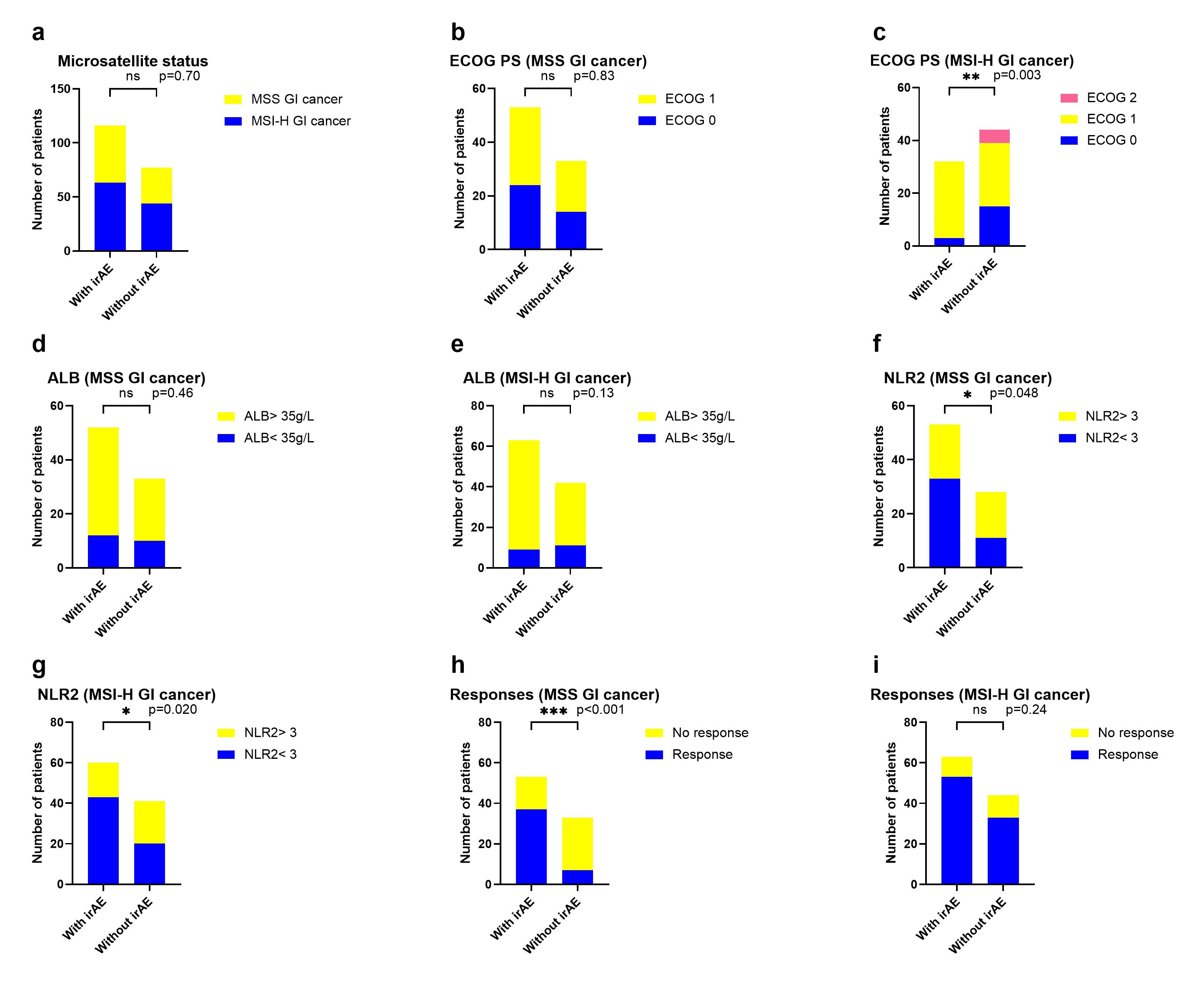

Supplement: Supplementary file 1 [file cancers-14-03736-s001.zip › Figure S5.jpg]
